# Level-set based adaptive-active contour segmentation technique with long short-term memory for diabetic retinopathy classification

**DOI:** 10.3389/fbioe.2023.1286966

**Published:** 2023-12-19

**Authors:** Ashok Bhansali, Rajkumar Patra, Mohamed Abouhawwash, S. S. Askar, Mohan Awasthy, K. B. V. Brahma Rao

**Affiliations:** ^1^ Deptartment of Computer Engineering and Applications, GLA University, Mathura, India; ^2^ Department of Computer Science and Engineering, CMR Technical Campus, Hyderabad, India; ^3^ Department of Computational Mathematics, Science and Engineering (CMSE), College of Engineering, Michigan State University, East Lansing, MI, United States; ^4^ Department of Mathematics, Faculty of Science, Mansoura University, Mansoura, Egypt; ^5^ Department of Statistics and Operations Research, College of Science, King Saud University, Riyadh, Saudi Arabia; ^6^ Department of Engineering and Technology, Bharati Vidyapeeth Deemed to be University, Navi Mumbai, India; ^7^ Department of Computer Science and Engineering, Koneru Lakshmaiah Education Foundation, Guntur, India

**Keywords:** adaptive-active counter model, diabetic retinopathy, gray level co-occurrence matrix, level set method with improved boundary indicator function, long short term memory

## Abstract

Diabetic Retinopathy (DR) is a major type of eye defect that is caused by abnormalities in the blood vessels within the retinal tissue. Early detection by automatic approach using modern methodologies helps prevent consequences like vision loss. So, this research has developed an effective segmentation approach known as Level-set Based Adaptive-active Contour Segmentation (LBACS) to segment the images by improving the boundary conditions and detecting the edges using Level Set Method with Improved Boundary Indicator Function (LSMIBIF) and Adaptive-Active Counter Model (AACM). For evaluating the DR system, the information is collected from the publically available datasets named as Indian Diabetic Retinopathy Image Dataset (IDRiD) and Diabetic Retinopathy Database 1 (DIARETDB 1). Then the collected images are pre-processed using a Gaussian filter, edge detection sharpening, Contrast enhancement, and Luminosity enhancement to eliminate the noises/interferences, and data imbalance that exists in the available dataset. After that, the noise-free data are processed for segmentation by using the Level set-based active contour segmentation technique. Then, the segmented images are given to the feature extraction stage where Gray Level Co-occurrence Matrix (GLCM), Local ternary, and binary patterns are employed to extract the features from the segmented image. Finally, extracted features are given as input to the classification stage where Long Short-Term Memory (LSTM) is utilized to categorize various classes of DR. The result analysis evidently shows that the proposed LBACS-LSTM achieved better results in overall metrics. The accuracy of the proposed LBACS-LSTM for IDRiD and DIARETDB 1 datasets is 99.43% and 97.39%, respectively which is comparably higher than the existing approaches such as Three-dimensional semantic model, Delimiting Segmentation Approach Using Knowledge Learning (DSA-KL), K-Nearest Neighbor (KNN), Computer aided method and Chronological Tunicate Swarm Algorithm with Stacked Auto Encoder (CTSA-SAE).

## 1 Introduction

Diabetic Retinopathy (DR) is one of the most common types of retinal vascular complication of diabetes mellitus manifested by elevated blood sugar which severely affects the blood vessels of retinal tissue (Garifullin et al., 2021; Huang et al., 2022). DR is a complex condition that can result in vision loss, and projections indicate that by 2040, approximately 600 million individuals will be affected by it with one-third of them experiencing diabetic retinopathy. Diabetic microvascular disease leads to DR which is classified into three classes such as blood vessel rupture, hemorrhage, and obstruction of blood vessels. Moreover, DR is categorized into five stages such as normal, mild, moderate, severe, and proliferative (Hasan et al., 2021; Xu et al., 2021; Pundikal and Holi, 2022; Maaliw et al., 2023) based on the severity of disease. Regular screening is required to aid in early DR identification, and early DR discovery will allow for thorough monitoring of the DR progression rate, which can be remarkably quick from early to high risk. By detecting and treating DR abnormalities early on, it is possible to prevent 95% of premature, irreversible visual damage as well as subsequent recurrences. At the initial stage, DR does not show any symptoms or minor vision impairment in the body parts. The symptoms of DR include blurred or color vision impairment and dark strings, which occur in the float of the patient’s vision (Sule, 2022; Ullah et al., 2023). DR can be diagnosed with the help of a laser or through a surgical procedure known as vitrectomy which inhibits the changes and helps to retain the vision. Non-Proliferative Diabetic Retinopathy (NPDR) leads to retinal swelling and minute blood vessel leaks (Kadan and Subbian, 2021; Nikoloulopoulou et al., 2023). The extreme phase in DR is referred to as Proliferative Diabetic Retinopathy (PDR) which damages the inner tissues of the retina and blocks the flow of blood (Guo and Peng, 2022; Li et al., 2022; Yan et al., 2022; Sundaram et al., 2023).

The formation of scar tissue has the maximum probability of affecting the central and peripheral vision. The manual diagnosis of DR is a time-consuming method, so computer-aided diagnosis gains attention among the ophthalmologist (Nallasivan et al., 2021; Ali et al., 2023). The hard exudates rupture the fatty blood vessels and worsen the condition of diabetic retinopathy. These hard exudates are yellow with different sizes and shapes. Since diabetic retinopathy is considered as a major concern which affects the people, relies a precise segmentation must be employed to detect and classify the type of diabetic retinopathy (Atli and Gedik, 2021; Udayaraju et al., 2023). The researchers were focusing on traditional image processing approaches such as morphological operations and threshold segmentation approaches. The existing researches are limited by heavy dependence of design level and the traditional lesion segmentation approaches. However, the existing research based on DR classification has not attained granularity while distinguishing PDR (Alam et al., 2023). Moreover, deep learning techniques are used in various applications related to segmentation and classification. Although deep learning techniques hold enormous promise for applications involving clinical imaging, the present techniques call for a greater volume of trained labelled data. Training datasets frequently include thousands of high-quality labelled photos, which are expensive to acquire and unavailable for rare conditions, but are necessary to achieve better outcomes. In addition to making more data available, enhancing current techniques to get the same results with less data offers another potential remedy for these restrictions. Precise segmentation has a significant stage in classifying DR with better categorization accuracy (Jebaseeli et al., 2019; Atwany et al., 2022; Chen et al., 2023). The existing approaches faced problems related to poor segmentation accuracy due to its incapability in detecting the boundaries of the image. Moreover, the edges of the images are not considered as the major part while segmentation which affects the classification accuracy of the model. So in this research, an effective segmentation approach by considering the boundary which makes the classification process easier and aids in better classification accuracy.

The significant contributions of this research are specified as follows:

1) In this research, the raw data obtained from IDRiD and DIARETDB 1 is pre-processed by noise removal using Gaussian filter, enhancing the color and luminosities of the image. The pre-processed image was used in the process of segmentation to improve the image quality.
2) Secondly, segmentation was performed before the stage of feature extraction and classification. The stage of segmentation plays an important role in analyzing the retinal fundus. So, this research introduced LBACS technique to segment the DR images.
3) The LBACS is comprised with LSMIBIF and AACM which effectively overcome the limitations of existing approaches in segmenting the boundaries and edges. The proposed approach effectively segments the image into partitions and detect the DR in every individual segments using LSMIBIF and AACM respectively.
4) The features are extracted using GLCM, LTP and HOG, which extract the features based on gradient and intensity of the image. Finally, the classification is performed with the help of LSTM to classify the type of diabetic retinopathy.

The rest of the paper is structured as follows, Section 2 presents the related work of this research and Section 3 presents the proposed method of this research. The results are presented in Section 4, while the Section 5 presents the overall conclusion of this research.

## 2 Related works

In this section, recent researches based on diabetic retinopathy segmentation and classification techniques are discussed. This section was classified based on the usage of deep learning and machine learning approaches in classifying the diabetic retinopathy.

### 2.1 DR classification using deep learning methods

Rachapudi et al. (2023) developed an optimized classification method using the Deep Neural Network and Butterfly Optimization Algorithm (DNN-BOA). After the data acquisition and pre-processing, the blood vessels were removed with the help of Gray Level thresholding, and segmentation was performed with the help of Modified Expectation Maximization (MEM). The feature extraction was performed using the GLCM and finally, categorization was performed using DNN-BOA. However, the features extracted by the suggested approach had higher dimensionalities which enhanced the processing time. Dayana and Emmanuel (2022b) introduced an efficient and optimized deep neural network along with a Chronological Tunicate Swarm Algorithm (CTSA) to categorize the severity of diabetic retinopathy. The U-Net and the fuzzy C-means hybrid entropy model were utilized to segment the optic disc and blood vasculatures. After this, the Gabor filter was used to identify the region of lesions and finally, the classification was performed with the help of a Stacked Autoencoder with CTSA on chronologic concept. However, the suggested approach faced misclassification issues while discerning the mild class DR.

Mondal et al. (2023) introduced an Ensemble Deep Learning Technique to Detect and categorize diabetic retinopathy. In this research, two deep learning approaches such as DenseNet101 and ResNeXt models were used for classification stage. The histogram equalization was performed using CLAHE and data augmentation was performed with the help of GAN based augmentation technique. However, the suggested approach did not perform a feature map for the whole input data. Dayana et al. (2023) introduced an improved classification method based on an optimization approach to grade the severity of the image. The dilated convolution-based spatial attention U-Net was used in optic disc segmentation and the entropy-based hybrid approach was used in the process of segmenting blood vessels. The extraction of features took place using a layered fusion network and the classification was performed with the help of a Refined Deep Residual Network (RDRN) and Tunicate Swarm Spider Monkey Optimization (TSSMO) algorithm. However, misclassification occurred while classifying the mild lesions with a dark background and overlying visuals.

Beham and Thanikaiselvan (2023) introduced an optimized deep-learning approach for automated retinopathy detection. The suggested approach introduced an Inception V3 model with a customized Convolutional Neural Network (CNN) with the Population-Based Incremental Learning (PBIL) algorithm referred to as PBIL-CNN. The PBIL-CNN effectively detected the DR from the color fundus images and helped in the process of classifying diabetic retinopathy. The suggested approach considered the feasible fitness function to distinguish the parameters of CNN. However, the PBIL-CNN exhibited complexities while computing the deeper samples such as dilation of pupils. Shanthini et al. (2021) introduced a Segmentation Convolutional Neural Network (S-CNN) to detect diabetic retinopathy. The S-CNN performed threshold-based segmentation to categorize the foreground and the background of the retinal image. The segmentation was performed with the pixel-based segmentation approach. The layers were accessed using the two-layer CNN which mitigated the rate of false positives during DR classification. However, the noise reduction algorithm used in pre-processing reduced the dark information fine contrasts. Özçelik and Altan (2023) developed a robust AI model that makes use of two-dimensional stationary wavelet transform (2D-SWT) and fractal analysis to handle the crucial problem of early identification in diabetic retinopathy. Validated by 10-fold cross-validation, the model used a recurrent neural network-long short-term memory (RNN-LSTM) architecture for classification and shown remarkable performance in diagnosing all stages of DR with low computational cost. However, the suggested framework faced complexities while reducing the dimensionality of the features.

Usman et al. (2023) developed an approach of using pre-trained CNNs such as ResNet50, ResNet152, and SqueezeNet1 to concentrate on color Fundus Photographs (CFPs) dimensionality reduction and data preparation. This study was focused to address issues of existing screening techniques which were frequently underutilized, delayed diagnosis and impaired vision. This approach also introduced a Deep Learning Multi-Label Feature Extraction and Classification (ML-FEC) model. The results have shown that ResNet152 achieved a low Hamming loss indicating that it could be useful in large-scale DR screening applications. Alajlan and Razaque. (2023) have introduced Egret Swarm Optimization based Hybrid Gated Recurrent Unit (ESOA-HGRU) for classifying DR. Initially, the input samples are pre-processed using the data augmentation approach and partitioned as training and testing data. The hybrid mask region was optimized with the help of Egret Swarm Optimization to diminish the loss of classifier. Finally, the classification was performed with the help of ESOA-HGRU. However, the suggested approach exhibits poor performance for asymmetric datasets. Math and Fatima (2021) have introduced an adaptive approach to segment and classify the DR images. Initially, the data is pre-processed using normalization method and the scaling is performed to equalize the contrast and illumination. After this, the CNN based segment level classifier is used to evaluate the possibilities of DR. Next to the stage of segmentation, the global aggregation is performed to integrate the segment level DR images and classification is performed with the help of CNN. However, the usage of CNN requires more number of labelled data.

### 2.2 DR classification using machine learning methods

Shaukat et al. (2022) introduced a three-dimensional semantic model to segment the diabetic retinopathy lesions. The pre-processed input data was fed into the pre-trained Xception model, and the extracted features were segmented with the help of Deeplabv3. After this, the feature selection was performed with the help of the Marine Predictor Algorithm (MPA). Finally, the classification of DR takes place with the help of a neural network and K-Nearest Neighbor classifier. However, the classification accuracy of the suggested approach was diminished for varying sizes of DR lesions. Kaur and Kaur (2022) developed an automated diagnosis of diabetic retinopathy based on segmentation and classification using the K-Nearest Neighbor (KNN) classifier. In the stage of pre-processing, the unwanted pixels were removed and 2D discrete wavelet decomposition was applied to extract the boundaries of blood vessels. Moreover, an adaptive thresholding approach was used to detect the statistical and geometrical location of the lesions. Finally, the KNN classifier was used in the process of classifying the DR lesions. The suggested approach lacked efficiency while evaluating the high-dimensional features. Jaskirat et al. (2023) developed an automated detection and segmentation approach to detect and segment the exudates of the acquired image retina based on digital fundus images. The suggested approach enhanced the quality of the retinal fundus image and removed the false exudate pixels. Additionally, the mass screening process filtered the unwanted portion that was not related to diabetic retinopathy. However, the evaluation of the suggested approach was limited with a minimal number of retinal images.

Sandhya et al. (2022) introduced a Delimiting Segmentation Approach Using Knowledge Learning (DS-KL) to enhance the accuracy of diabetic retinopathy. The suggested approach was based on the histogram variation classification that detected the exudate regions. The input images were obtained from the variation that took place from the histogram variations that help to detect the exudate regions. The segmentation approach discriminated the affected regions by delimiting the pixel boundaries. However, the suggested approach faced classification errors while categorizing the complex features of distributed image segments. Da Rocha et al. (2022) developed a novel approach of using the VGG16 neural network to address critical issues of diabetic retinopathy. The objective of this study was to create a fifth class (class 5) for low-quality digital retinal images from the DDR, EyePACS/Kaggle, and IDRiD databases in addition to classifying diabetic retinopathy into five categories. The methodology included image size modification, data cleaning, augmentation, class balance, and hyperparameter tuning. This method improved the standard of diagnosis and treatment for diabetic retinopathy. The Table 1 depicted below presents the outcome of key characteristics to highlight the constraints and proposed solution of the existing researches.

**TABLE 1:** Comparison of existing methodology, their advantage and limitation in classifying DR.

Author	Methodology	Advantage	Limitation
Rachapudi et al	DNN-BOA used in the process of classifying diabetic retinopathy	Higher dimensions helped in enhancing the processing time	However, the features extracted by the suggested approach had higher dimensionalities which affects the overall classification approach
Dayana and Emmanuel et al	An efficient and optimized deep neural network along with a Chronological Tunicate Swarm Algorithm (CTSA) to categorize the severity of diabetic retinopathy	Stacked Autoencoder along with CTSA helps in classification of DR in chronologic order	Misclassification problems in identifying the moderate class DR
Mondal et al	An Ensemble Deep Learning Techniques such as DenseNet101 and ResNet for classification of DR	The equalization of histogram using CLAHE performs contrast amplification for each neighboring pixels	The measure of feature classes was difficult as feature map of input data was not performed
Dayana et al	Refined Deep Residual Network (RDRN) and Tunicate Swarm Spider Monkey Optimization (TSSMO) algorithm	The segmentation of optic disc and blood vasculatures helps in the process of extracting the appropriate features	Misclassification while classifying the mild lesions with dark background and overlying visuals
Beham and Thanikaiselvan	An optimized deep learning approach Convolutional Neural Network (CNN) with the Population-Based Incremental Learning (PBIL)	The parameters of CNN were easily differentiated from the fitness function	Complexities while computing the deeper samples such as dilation of pupils
Shanthini et al	Segmentation Convolutional Neural Network (S-CNN) to detect diabetic retinopathy	The two-layer CNN effectively reduced false positive rate during DR classification	The noise reduction algorithm used in pre-processing reduced the dark information fine contrasts
Yusuf Bahri Özçelik et al	Robust AI model using two-dimensional stationary wavelet transform (2D-SWT) and also RNN-LSTM for classification of DR	The 2D-SWT model performs pixel wise segmentation which minimize the complexities in extracting the features	The suggested framework does not work for segmenting the deep samples such as dilated pupils and the discs
Tiwalade Modupe Usman et al	Principal component analysis multi-label feature extraction and classification using pre-trained CNN and SqueezeNet1 to concentrate on color Fundus Photographs (CFPs) dimensionality reduction and data preparation	The usage of CNN and squeeze net 1 minimize the hamming loss and aids in better classification results	The complexities occurs while classifying the complex features such as deep edges of pupil and iris
Abrar M. Alajlan and Abdul Razaque	Egret Swarm Optimization based Hybrid Gated Recurrent Unit (ESOA-HGRU) for classifying DR	ESOA optimize the loss function and helps to classify the DR using HGRU	However, the high complex features present at the edges of the image were unrecognized
Laxmi Math and Ruksar Fatima	An adaptive approach using CNN based segment level classifier to evaluate the possibilities of DR	The global aggregation approach was used to integrate the segmented image to obtain better classification results	The suggested approach does not works well for unlabeled datasets
Shaukat et al	A three-dimensional semantic model to segment the diabetic retinopathy lesions and KNN for classification	The MPA utilized in the feature selection performs an in-depth search to select the relevant features	Low classification accuracy for varied sizes of DR lesions
Kaur and Kaur et al	An automated diagnosis of diabetic retinopathy based on segmentation and classification using the KNN classifier	2D discrete wavelet decomposition works till the boundaries of blood vessels which makes the classification easier	Efficiency of the model is low while evaluating the high dimensional features
Jaskirat et al	An automated detection and segmentation approach to detect and segment the exudates of the acquired image retina based on digital fundus images	Enhanced quality of retina fundus images and removed eye false exudate pixels. The unwanted portions which are not related to DR were removed through mass screening process	Limited number of retinal images resulted in the minimal performance of the model
Sandhya et al	Delimiting Segmentation Approach Using Knowledge Learning (DS-KL) to enhance the accuracy of diabetic retinopathy	The histogram variations helped to detect the exudate regions and also the effected regions were eliminated by easily by narrowing the pixel boundaries	Classification errors while categorizing the complex features of distributed image segments
Douglas Abreu da Rocha et al	VGG16 Neural network for classification of DR	The presence of max pooling layer and the dense layer evaluate the selected features and helps in effective DR classification	However, the embedded image visuals of the DR images cannot be detected due to absence of in-depth analysis

#### 2.2.1 Scientific contribution

The aforementioned existing segmentation approaches did not consider the edges and the boundary conditions while segmenting the retinal images. When the segmentation approach does not consider the edges and the boundaries, the segmentation accuracy is less with improper boundaries which will affect the overall performance of the model. So, this research introduces an effective segmentation approach using LBACS and classification using LSTM to categorize various classes of DR with better accuracy. Moreover, LSMIBIF and AACM in LBACS allocate an improved boundary condition and detect the edges of the pre-processed image respectively. Thus, the proposed LBACS-LSTM provides better results in both segmentation and classification of diabetic retinopathy.

## 3 LBACS-LSTM method

**FIGURE 1:**
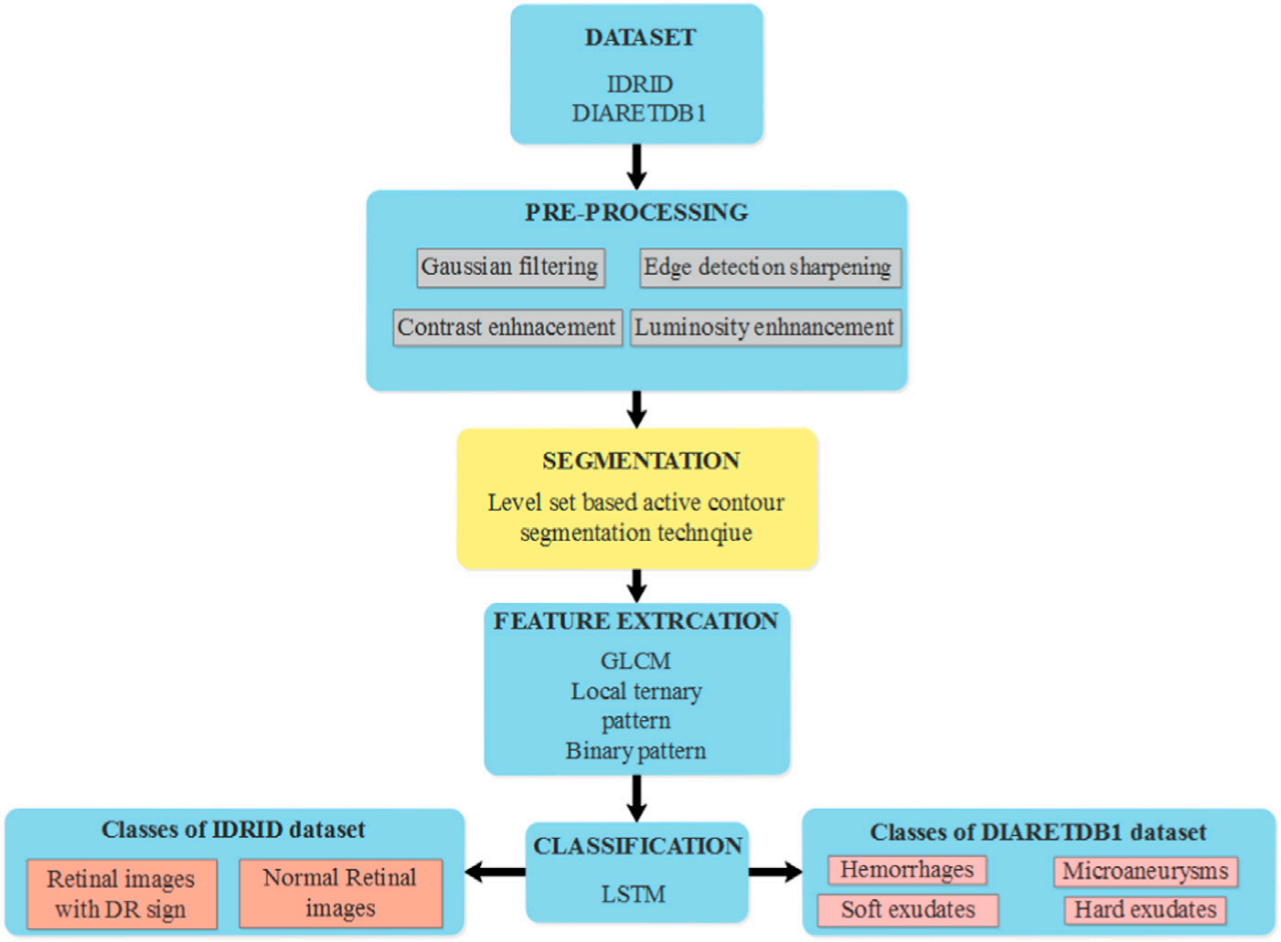
The overall process involved in the classification of diabetic retinopathy using LBACS-LSTM.

In this research, effective segmentation and classification are performed with the help of LBACS, and the classification is performed with the help of LSTM classifier. Diabetic retinopathy is categorized by employing the following stages; initially, the data is acquired from publicly available datasets such as IDRID and DIARETDB1 and the pre-processing is performed with Gaussian filtering, edge detection sharpening, enhancement of contrast and luminosity. Then the pre-processed output is fed into the stage of segmentation where an effective segmentation is performed using the proposed LBACS. The segmented output is fed into the stage of feature extraction and finally, the categorization is performed using the LSTM classifier. Figure 1 presents the block diagram of the overall process involved in classifying diabetic retinopathy.

### 3.1 Data acquisition

In this research, the data is obtained from two publicly available datasets as Indian Diabetic Retinopathy Image Dataset (IDRiD) (Porwal et al., 2018) and Diabetic Retinopathy Database 1 (DIARETDB 1) (Kaggle, 2023). This section presents a brief description of those two datasets.

#### 3.1.1 IDRiD

**FIGURE 2:**
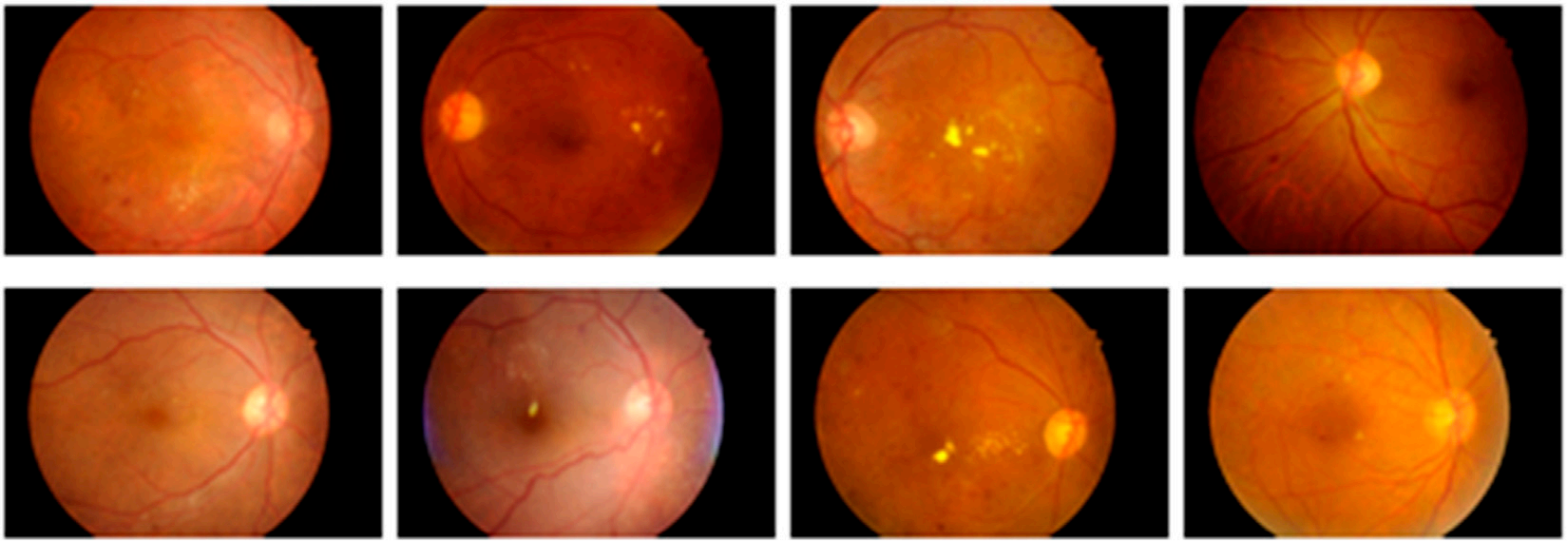
Sample images of IDRiD dataset.

It is a publicly accessible dataset that can be downloaded from IEEE data port repository. This dataset is comprised of fundus images obtained from Indian eye clinics using the Kowa VX fundus camera. The captured IDRiD dataset has a 50-degree field view with a resolution of 4288 × 2848. Figure 2 presents the sample image obtained from the IDRiD dataset.

#### 3.1.2 DIARETDB 1

**FIGURE 3:**
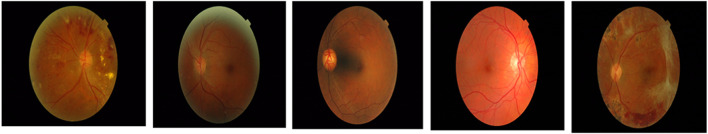
Sample images obtained from DIARETDB 1.

This is a type of publicly available dataset that consists of 89 color fundus images of which 84 have mild NPDR signs, and the rest of the 5 images are normal images. Specifically, 41 images are bright lesions and 45 images are darker ones. The resolution of the pixels present in this dataset is 1500 × 1152 and the angle of vision is 50°. Among 89 images, 28 images are used for training and the remaining 61 images are for testing. Figure 3 shows the sample images in the DIARETDB1 dataset.

### 3.2 Data pre-processing

After the stage of data acquisition, the raw data is pre-processed to get a pre-processed output without any noise. In this research, the pre-processing is performed with the help of Gaussian filtering, edge detection and sharpening, and enhancement of color and luminosity. In this section, the process involved in the aforementioned pre-processing techniques are described as follows:

#### 3.2.1 Gaussian filtering

The fundus image is comprised of three bands of red, green, and blue. The exudates look brighter in color compared to red and blue channels. The Gaussian filter (González-Ruiz et al., 2023) smoothens the average value of neighboring pixels and removes noise, and the high-frequency constituents present in the image are calculated based on the Eq. 1 as follows:

$$I_{g}\left(x,y\right)=I_{g}\left(x,y\right)\times g\left(x,y\right)$$ (1)

Where the Gaussian function is represented as $g\left(x,y\right)$ , the green channel component and the Gaussian noise is represented as $I_{g}\left(x,y\right)$ and $I_{g}\left(x,y\right)$ respectively.

#### 3.2.2 Edge enhancement

It is one of the image processing techniques that improvise the edge contrast of the image to enhance sharpness of the image. This process creates a subtle bright and dark highlight of edges in the image and makes it look more defined.

#### 3.2.3 Enhancement of color and luminosity

**FIGURE 4:**
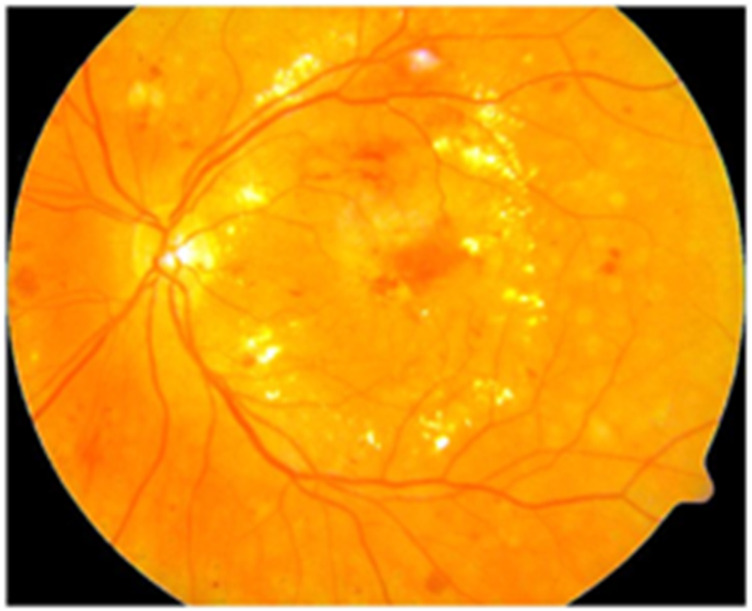
Sample image obtained after enhancing the color and luminosity.

After edge enhancement, the color and luminosity of the image are enhanced with the help of Contrast Limited Adaptive Histogram Equalization (CLAHE). The CLAHE (Chandni et al., 2022) creates a realistic form of the image by improvising the color pattern and luminosity by histogram equalization. The sample image obtained after enhancing the color and luminosity is represented in Figure 4 as follows.

### 3.3 Segmentation

After the stage of pre-processing, the pre-processed output is fed into the stage of segmentation which is performed with the help of Level-set Based Adaptive-active Contour Segmentation (LBACS) technique. The following section describes the process involved in segmentation using the proposed LBACS which is the improvisation made with boundary indicator function in level set function, and adaptive method in active contour method. The segmentation efficiency is enhanced with the help of improved boundary conditions and an adaptive active counter to determine the edges of the pre-processed image. The detailed information related to the Level Set Method (LSM), the Level Set Method with Improved Boundary Indicator Function (LSMIBIF), and Adaptive-Active counter models are described in the sub-sections below.

#### 3.3.1 Level set method (LSM)

The contour has a zero level set of time-dependent Level Set Function (LSF) and assumes that LSF considers the negative value inside zero level contour and positive values at the outside counter. The Euler’s equation for Distance Regularized Level Set Evolution (DRLSE) is represented in Eq. 2 as follows:

$$\phi_{t}=\mu \;\mathrm{div}\left(d_{p}\left|\nabla_{\phi }\right|\nabla_{\phi }\right)+\lambda \delta_{\varepsilon }\left(\phi \right)\mathrm{div}\left(\frac{g^{\prime }\left|\nabla_{\phi }\right|}{\alpha g^{\prime }\delta_{\varepsilon }\left(\phi \right)}\right)$$ (2)

The edge indicator function of DRLSE is denoted as $g^{\prime }$ which is represented in Eq. 3 as follows:

$$g^{\prime }=\frac{1}{1+{\left|f\right|}^{2}}$$ (3)

The weighted coefficient values are represented as μ,λ,α, and the double well potential distance for regularization is represented as $d_{p}.$ The differentiation of the of initialization process is represented as $\nabla_{\phi }.$ The hat function along variable support is represented as $\delta_{\varepsilon }$ . The existing LSM eliminates the need for re-initialization and utilizes the binary step function to initialize level set factor. However, the edge detector function in the existing level set method fails in detecting the accurate boundaries due to the challenges in illumination and low intensity issues. So, this research introduces LSMIBIF to set the appropriate boundary for the pre-processed image.

#### 3.3.2 Level set method with Improved Boundary Indicator Function (LSMIBIF)

The issues rely on the existing LSM being overwhelmed using the proposed LSIMIBIF. The improvisation is made in the boundary indicator function which segments the diabetic retinopathy in patients. The consideration of boundary function A counter is combined with a zero level set of level set factor ϕ , and the level set factor is determined based on $\Omega_{in}$ . The $\Omega_{0}$ is known as the zero-level set where $\left(\phi =0\right)$ , $\Omega_{in}$ is the domain inside $\Omega_{0}$ and the domain outside $\Omega_{0}$ is represented as $\Omega_{out}$ . The energy function $E\left(\phi \right)$ is represented in Eq. 4 as follows:

$$E\left(\phi \right)=\varepsilon_{img}\left(\phi ,g_{\rho }\right)+\varepsilon_{reg}\left(\phi ,g_{\rho }\right)$$ (4)

Where the external energy which is determined with the help of image attribute is denoted as $\varepsilon_{img}$ and the regularization term which defines the utilized energy is represented as a constraint of LSM. The improved edge detector is represented in Eq. 5 which is improvised in the existing boundary condition represented in Eq. 3.

$$g_{\rho }=\frac{1}{1+\frac{1}{2}\left(1-{\left|1-\nabla I_{\sigma }\right|}^{2}/\rho^{2}\left({\left|1-\nabla I_{\sigma }\right|}^{2}/\rho^{2}\right.\right)}$$ (5)

Where the image smoothening performed with the Gaussian filter using standard deviation σ is represented as $I_{\sigma }.$ The ρ presents the boundary threshold function based on the standard deviation S and the value of the boundary threshold is evaluated using the Eq. 6 as follows:

$$\rho \left(I\right)=\frac{1+\sqrt{S\left(I_{\sigma }\right)}}{3}$$ (6)

The improved boundary indicator is used to exhibit the expression related to gradient descent value which is presented in Eq. 7 with three different parts. The first part is based on the regularization term that excludes the process of re-initialization. The second part is based on zero level set approach which offers long term driving for the boundaries of the target. The third part of the equation is utilized to enhance the region among the neighboring targets and the evolution rate.

$$\frac{\partial \phi }{\partial t}=udiv(d_{p}(d_{p}\left|\nabla_{\phi }\right|\nabla_{\phi }+\lambda \delta_{\varepsilon }\left(\phi \right)\mathrm{div}\left(g_{\rho }\frac{\nabla_{\phi }}{\left|\nabla_{\phi }\right|}\right)+\left(\alpha g_{\rho }+mY\right)\delta_{\varepsilon }\left(\phi \right))$$ (7)

Where the weighed co-efficient which evaluates each parameter is represented as u,λ , α and m. The difference in level set factor with respect to time is represented as $\frac{\partial \phi }{\partial t}$ . The divergence is evaluated using the following parameter is represented as $\mathrm{div}\left(.\right).$ The gradient operator of the LSF gradient is represented as ∇ and the Dirac function is represented as $\delta_{\varepsilon }$ which is represented in Eq. 8 as follows:

$$\delta_{\varepsilon }\left(\phi \right)=\left\{\begin{array}{c} \frac{1}{2\varepsilon }\left(1+\cos\left(\frac{\pi \phi }{\varepsilon }\right)\right)\left|\phi \right|\leq \varepsilon \\ 0\left|\phi \right|>\varepsilon \end{array}\right.$$ (8)

Where the obtained Heaviside function is represented as $\delta_{\varepsilon }$ . The Dirac function is only useful when used in conjunction with the integral. Because this Dirac function acquires the boundary near the zero-level set, the line integral of the boundary indicator with the active contour is determined by adding the Dirac function. The external energy is then obtained and used to calculate the contour as part of the evolution stimulus. The parameter employed in the Dirac function affects the function. The value of ε needs to be larger to enhance the capturing range of contour, but the higher contour range may affect the prediction accuracy. So, the value of ε is assigned as 1.5.

#### 3.3.3 Adaptive-Active Counter Model (AACM)

**FIGURE 5:**
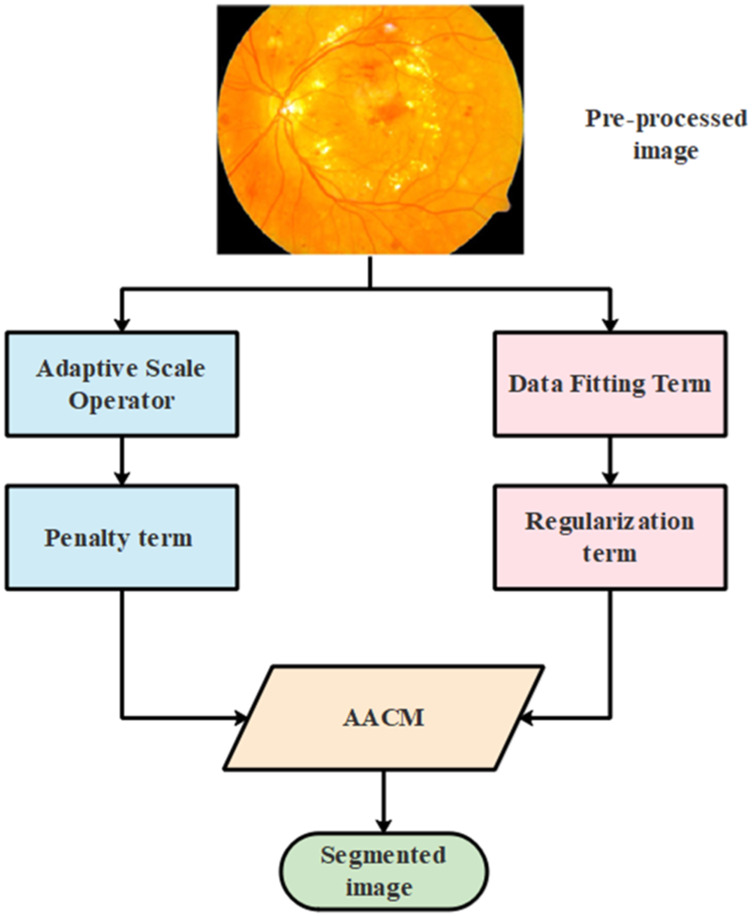
The process involved in segmentation using AACM.

AACM is used in the second stage of segmentation where the edges are detected to minimize the size of visual data and aid in better classification. The general architectural diagram of the process involved in segmenting the image using AACM is represented in Figure 5 as follows:

The energy function of the suggested AACM is comprised with two terms such as data fitting and regularization term which is represented in Equation 9 as follows:

$$E_{AACM}=E_{R}+E_{D}$$ (9)

Where the term related to data fitting and regularization is represented as $E_{R}$ and $E_{D}$ respectively.

##### 3.3.3.1 Data fitting term

The entropy value of the Shannon information to the image domain and the proposed image entropy value is represented in Eq. 10 as follows:

$$E_{img}=-{\int \int }_{\Omega_{x}}P_{y,\Omega_{x}}\log P_{y,\Omega_{x}}dxdy$$ (10)

Where the intensity of distributing small circular neighborhoods is represented as $P_{y,\Omega_{x}}$ and the equation of the adaptive scale operator is denoted in Eq. 11 as follows:

$$ASO=\left\{\begin{array}{c} \frac{1}{2\pi \sigma }\exp\left(-\frac{d_{x}}{{2\sigma }^{2}}\right),d_{x}\leq \rho \\ 0,d_{x}>\rho \end{array}\right.$$ (11)

Where the Euclidean distance from point y to point x is represented as $d_{x}$ and the radius which is responsible to control the distance of the radius is represented as ρ , represented in Eq. 12 as follows:

$$\rho =\theta \;mean\left(1-\frac{E_{img}-\min\left(E_{img}\right)}{\max\left(E_{img}\right)-\min\left(E_{img}\right)}\right)$$ (12)

Where the mean operator and the factor related to normalization are represented as mean and θ respectively. The images with severe inhomogeneous intensity have blurry regions with edges and the sub-region cannot be discriminated with the help of human vision. To overcome this issue, the bias field estimation term is described based on the membership function for every individual pixel value. The improved bias field estimation term is represented in Eq. 13 as follows:

$${\hat{I}}_{\phi_{i}}\left(y\right)=b\left(x\right)u_{i}^{m}\left(x\right)I_{\phi_{i}}\left(y\right)+n\left(y\right)$$ (13)

Where the value of fitting intensity is represented as ${\hat{I}}_{\phi_{i}}\left(y\right)$ and the value of real intensity value is represented as $I_{\phi_{i}}\left(y\right).$ The dependent membership function is represented as $u_{i}^{m}\left(x\right)$ which lies in the point y and the fuzzy index value m. The varying bias field related to inhomogeneous intensity is represented as $b\left(x\right).$ The additive noise present in the Gaussian distribution function is represented as $n\left(y\right)$ . Based on the semi-Naïve Bayes classifier, the proposed AACM model is defined in Eq. 14 as follows:

$$p\left(y\epsilon \Phi_{i}|I(y\right))=\frac{\prod_{i=1}^{N}p\left(y\epsilon \Phi_{i}\right)p\left(I\left(y\right)|y\epsilon \Phi_{i},u_{i}^{m}\left(y\right)\right)}{p\left(I\left(y\right)\right)}$$ (14)

Where the respective region area is represented as A and the Gaussian distribution function with the final energy fitting term $E^{D}$ is represented in Eq. 15 as follows:

$$E^{D}=\sum_{i=1}^{N}{\int \int }_{\phi_{i}}-k_{p}^{ASO}\left(d_{x}\right)\left[\ln\frac{1}{\sqrt{2\pi \sigma_{i}\left(x\right)}}\exp\left(-\frac{{\left|I\left(y\right)-b\left(x\right)u_{i}^{m}\left(y\right)I_{i}\left(y\right)\right|}^{2}}{2{\sigma_{i}\left(x\right)}^{2}}\right)+\ln\left(\frac{A\left(\Phi_{i}\right)}{A\left(\Omega_{x}\right)}\right)\right]]dxdy$$ (15)

##### 3.3.3.2 Regularization term

The stabilized evolution and the smoothened level set function is represented as ϕ and the regularization term is considered as a significant component of AACM. In this research, the regularization term is developed with the help of penalty term and length regularization term which is represented in Eq. 16 as follows:

$$E^{R}=uL\left(\phi =0\right)+v_{R_{p}}\left(\phi \right)$$ (16)

Where the constant values which contribute to two terms are represented as u and v , the occurrence of tiny isolated sections in the final stage are represented as $L\left(\phi =0\right)$ and the penalty term to eradicate re-initialization and retain the signed function at curve evolution is represented in Eqs. 17, 18 respectively.

$$L\left(\phi =0\right)=\int_{\Omega }\delta \left(\left|\nabla \phi \right|\right)dxdy$$ (17)

$$R_{p}\left(\phi \right)=\int_{\Omega }p\left(\left|\nabla \phi \right|\right)dxdy$$ (18)

Where the Hamilton operator is represented as ∇ and the energy density function is represented as $p\left(\left|\nabla \phi \right|\right).$ The penalty term for the flow of gradient descent is represented in Eq. 19 as follows:

$$\frac{\partial \phi }{\partial t}=-\frac{\partial R_{p}}{\partial \phi }=\mathrm{div}\left(d_{p}\left(\left|\nabla \phi \right|\right)\nabla \phi \right)=\mathrm{div}\left(\frac{p^{\prime }\left(\left|\nabla \phi \right|\right)}{\left|\nabla \phi \right|}\nabla \phi \right)$$ (19)

Where the operator which is related to divergence is represented as div and the diffusion controlling rate is represented as $d_{p}\left(\left|\nabla \phi \right|\right)$ and the first order derivative is represented as $p\left(s\right)$ which lies among $\left[0,\infty \right)$ . Finally, by using the LSMIBIF and AACM, the pre-processed data is segmented boundary indicator function which segments the diabetic retinopathy in patients. The AACM detects the edge of the image obtained from LSMIBIF, also minimizes the size of visual data and aids in better classification.

### 3.4 Feature extraction

After segmentation, feature extraction is performed to select relevant or appropriate features that reduce the complexities while categorizing the diabetic retinopathy in patients. In this research, the features from the segmented output are extracted with the help of Local Ternary Pattern (LTP), Gray Level Co-occurrence Matrix (GLCM) and Histogram Oriented Gradients (HOG). The steps involved in extraction of features are presented as follows:

#### 3.4.1 Local Ternary Pattern

LTP (Dayana and Emmanuel, 2022b) is an improved version of Local Binary Pattern (LBP) which utilizes fixed threshold value to perform an effective extraction of binary pattern. LTP is computed using the Eq. 20 as follows:

$${LTP}_{P,r,\tau }=\sum_{i=0}^{P-1}s\left(Pi-Pc\right)\times 3^{i},s\left(x\right)=\left\{\begin{array}{c} 1\;x\geq \tau \\ 0\left|x\right|<\\ -1\;x\leq -\tau \end{array}\right.$$ (20)

Where the central pixel value is represented as Pc , the threshold value is represented as τ and the neighboring pixel value is represented as Pi . The feature dimensionalities are minimized by separating the individual ternary pattern into two parts such as positive and negative. After processing the segmented image using LTP, the two feature histogram values are acquired for every image.

#### 3.4.2 Gray level Co-occurrence matrix

The Gray Level Co-occurrence Matrix (GLCM) (Patel and Kashyap, 2023) is a kind of second-order statistical approach utilized in the process of analyzing image textures. The second order evaluates two-pixel pairs of the actual image. GLCM offers a probable combination of various gray level images. In this research, features such as contrast, energy, correlation, homogeneity and entropy are considered to extract features from segmented images. Moreover, GLCM evaluates interconnected pixels from the grayscale image of varying angles of $0^{o},45^{o},90^{o}$ and $135^{o}$ .

#### 3.4.3 Histogram Oriented Gradients

Histogram of Oriented Gradients (HOG) (Shaukat et al., 2023) are characterized by distributing the local intensity gradients and edge directions. HOG effectively captures and presents the deformations and the gradient orientation is represented in Eq. 21 as follows:

$$\theta_{XY}=\tan^{-1}\left(\frac{G_{Y}}{G_{X}}\right)$$ (21)

Where the gradient values of X and Y are denoted as $G_{X}$ and $G_{Y}$ respectively.

The features extracted using GLCM, LTP, and HOG are fed as input for the stage of diabetic retinopathy classification which is performed with the help of the Long Short Term Memory Classifier (LSTM) classifier.

### 3.5 Classification

**FIGURE 6:**
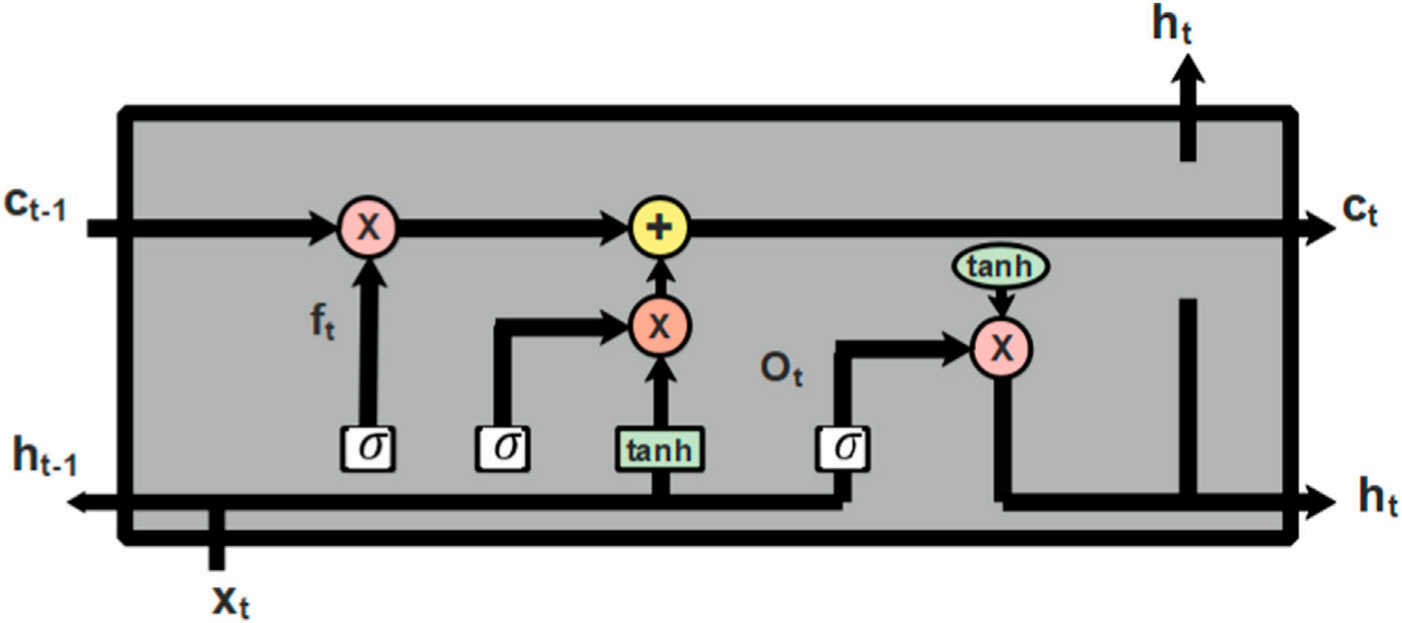
Architectural diagram of LSTM.

The features extracted from LTP, GLCM, and HOG are fed into the phase of classification which is performed using Long Short Term Memeory (LSTM) (Ewees et al., 2022), a deep learning classifier. The classification is the final stage where the extracted features are used to categorize the classes of diabetic retinopathy. LSTM is a kind of RNN that learns through long term dependencies. The architectural diagram of LSTM is represented in Figure 6 as follows:

Cell state is the central component of LSTM which can add or remove information from cells and selectively permit information to pass through the door mechanism to accomplish this. The forget gate, input gate, and output gate make up an LSTM. The input gate chooses what information to add to the cell state after the forget gate decides which information to remove from the cell state. The cell state can be updated once these two points have been established. The output gate regulates the network’s output. The process of node present in LSTM is described in Eqs. 22–27 as follows:

$$f_{t}=\sigma \left(W_{f}.\left[h_{t-1},x_{t}\right]+b_{f}\right)$$ (22)

$$i_{t}=\sigma \left(W_{i}.\left[h_{t-1},x_{t}\right]+b_{i}\right)$$ (23)

$${\tilde{C}}_{t}=\tanh\left(W_{C}.\left[h_{t-1},x_{t}\right]+b_{C}\right)$$ (24)

$$C_{t}=f_{t}*C_{t-1}+i_{t}*{\tilde{C}}_{t}$$ (25)

$$o_{t}=\sigma \left(W_{0}.\left[h_{t-1},x_{t}\right]+b_{0}\right)$$ (26)

$$h_{t}=o_{t}*\tanh\left(C_{t}\right)$$ (27)

Where, the hidden state of the prior layer is denoted as $h_{t-1}$ , input for the current layer is denoted as $x_{t}.$ The weight and biased state are denoted as Wandb respectively. The sigmoid function is denoted as σ and the output of the forget gate is denoted as $f_{t}.$ The output from the input gate is represented as $i_{t}$ and the intermediate temporary state is denoted as ${\tilde{C}}_{t}.$ The state of the cell present in the prior layer is denoted as $C_{t-1}$ and the state of the cell present in the next layer is denoted as $C_{t}.$ The output from the output gate and the hidden state of the succeeding layer is denoted as $o_{t}$ and $h_{t}$ respectively.

Computing the output of the input and output gate individually does not provide better performance so, the output from the input and output gate can be distinguished using the factor $1-f_{t}.$ This helps to improve the cell state for the next layer in the input and output gate, which is represented in Eq. 28 as follows:

$$C_{t}=f_{t}*C_{t-1}+\left(1-f_{t}\right)*{\tilde{C}}_{t}$$ (28)

The classification which is performed with the help of an LSTM classifier aids in better classification results due to its capability to select the image pattern for a longer time duration. The LSTM effectively classifies the various classes of diabetic retinopathy and helps the ophthalmologist to categorize the retina of the disease-affected patients.

## 4 Results and analysis

In this section, the information related to the experimental setup, performance metrics used to estimate the efficacy of the suggested approach, and results obtained through simulation analysis and comparative analysis are considered to evaluate the efficiency of the proposed approach.

### 4.1 Experimental setup

The proposed approach is simulated in Python software and the system is configured with specifications such as Windows 11 operating system, 16 GB of RAM and Intel i7 processor. The following python libraries are utilized in this research to evaluate the efficiency of the proposed approach. The data pre-processing is performed using the libraries such as NumPy, Pandas and Open CV. The libraries such as Tensor flow and Scikit-Learn are used to develop the deep learning classification model and result analysis respectively.

### 4.2 Evaluation metrics

The results obtained while evaluating the proposed approach are estimated by considering the performance metrics such as accuracy, sensitivity, specificity, precision, and dice co-efficient. The aforementioned performance metrics can be evaluated using the mathematical equations listed in Eqs 29–33.

**Accuracy:** It is defined as the fraction of total number of samples which are predicted accurately to the total number of samples.

$$Accuracy=\frac{TP+TN}{TN+TP+FN+FP}\times 100$$ (29)

**Sensitivity:** It is the type of performance metric used to predict the true positives and it is the ratio of true positives to the total number of true positives and the false negatives.

$$Sensitivity=\frac{TP}{TP+FN}\times 100$$ (30)

**Specificity:** It is ratio of proportion of true negatives to the sum of predicted false positives and true negatives

$$Specificity=\frac{TN}{TN+FP}\times 100$$ (31)

**Precision:** It is the ratio of proportion of true positive values to the sum of true positives and predicted false positives.

$$Precision=\frac{TP}{FP+TP}\times 100$$ (32)

$$Dice\;coefficient=\frac{2TP}{2TP+FP+FN}\times 100$$ (33)

Where, TP is true positive; TN is true negative; FP is false positive and FN is false negative.

### 4.3 Simulation results

In this section, the effectiveness of the suggested method is assessed based on segmentation and classification. The datasets such as IDRiD and DIARETDB 1 are utilized in evaluating the suggested method. The efficacy of the LBACS segmentation approach is related to the efficiency of state-of-art methods used for segmentation, and the efficacy of the LSTM classifier is assessed with some of the existing deep learning classifiers used in classifying diabetic retinopathy.

#### 4.3.1 Segmentation analysis

In this sub-section, the performance of LBACS is estimated with existing segmentation approaches such as Level set method (LSM), Active Contour model (ACM), Fuzzy C Means (FCM) algorithm and Region growing (RG). The segmentation accuracy is evaluated by considering the efficiency of the proposed approach while segmenting the pre-processed image. In other words, it is represented as ratio of correctly segmented pixels to the total number of pixels in the pre-processed image. The segmentation accuracy is mathematically represented in as follows:

Segmentation Accuracy=Total Number of PixelsNumber of Correctly Segmented Pixels (34)

The performance of the proposed segmentation approach is evaluated with the existing segmentation approach based on the accuracy value obtained at the time of segmentation and the value of dice co-efficient. Table 2 shows the results obtained while evaluating the proposed LBACS with the IDRiD dataset, while Table 3 shows the results obtained while evaluating the proposed LBACS with the DIARETDB 1 dataset.

**TABLE 2:** Evaluation of the proposed approach for the IDRiD dataset.

Segmentation models	Segmentation accuracy	Dice coefficient (%)
FCM	98.52	97.54
RG	96.79	96.11
LSM	96.09	95.88
ACM	97.24	97.03
LBACS	99.87	98.56

**TABLE 3:** Evaluation of proposed approach for DIARETDB 1 dataset.

Segmentation models	Segmentation accuracy	Dice coefficient (%)
FCM	95.78	95.23
RG	94.95	94.42
LSM	93.11	92.90
ACM	95.11	94.88
LBACS	96.89	96.56

The results from Table 1 and Table 2 demonstrate the analysis of the suggested approach for segmenting diabetic retinopathy by considering the data obtained from the IDRiD dataset and DIARETDB 1 dataset, respectively. The obtained outcomes depict that the suggested methodology achieved better segmentation accuracy of 99.87% for the IDRiD dataset and 96.89% for the DIARETDB1 dataset. These results are comparably higher than the existing methods, and this better result is due to the effectiveness of the suggested methodology by improving the boundary condition of the pre-processed image using LSIMBIF, while the AACM detects the edge of the image obtained from LSMIBIF, furthermore minimizing the size of visual data and aiding in better classification.

#### 4.3.2 Classification analysis

In this sub-section, the efficiency of the classifiers is assessed for the IDRiD and DIARETDB 1 datasets. Table 4 and Table 5 show the graphical representation for evaluation of classification performance for IDRiD and DIARETDB 1 datasets. The classification accuracy is the overall accuracy of the model while classifying the types of DR using the proposed segmentation approach. In other words, classification accuracy is defined as the accuracy score considered during classification task. It evaluates the proportion of correctly classified samples from the total number of samples and it can be mathematically represented in Eq. 35 as follows:

Classification accuracy=Total number of correctly classified samples (35)

**TABLE 4:** Evaluation of classification performance for IDRiD dataset.

Classifiers	Accuracy (%)	Sensitivity (%)	Specificity (%)	Precision (%)
RNN	95.23	95.96	96.80	96.82
CNN	93.64	93.23	93.87	94.48
GRU	97.45	98.36	98.10	97.74
GAN	94.89	95.23	95.47	94.99
LSTM	99.43	98.85	98.37	99.55

**TABLE 5:** Evaluation of classification performance for DIARETDB 1 dataset.

Classifiers	Accuracy (%)	Sensitivity (%)	Specificity (%)	Precision (%)
RNN	93.23	94.80	94.97	93.91
CNN	90.75	91.23	90.06	90.17
GRU	96.52	95.64	96.62	95.11
GAN	91.87	92.23	90.89	91.45
LSTM	97.39	96.55	98.91	97.12

**FIGURE 7:**
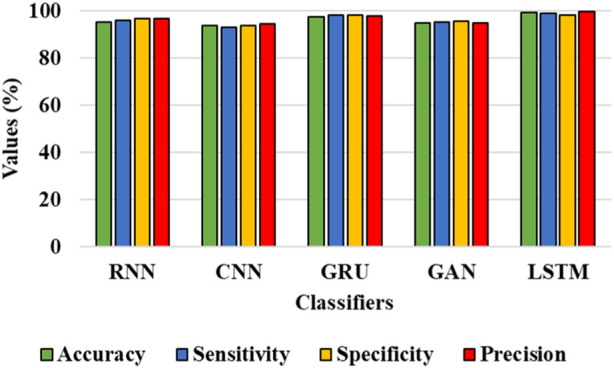
Graphical representation for the performance of the classifier for IDRiD dataset.

**FIGURE 8:**
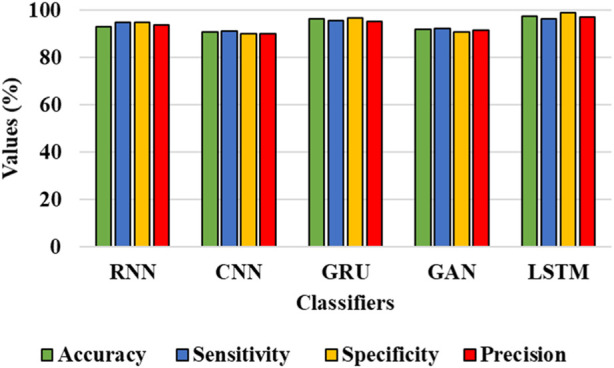
Graphical representation for the performance of the classifier for the DIARETDB 1 dataset.

The results from Table 4 and Table 5 show that the LSTM classifier used in this research obtains better classification results in overall metrics when compared with existing classification approaches. When LSTM is evaluated for the IDRiD dataset it obtains a classification accuracy of 99.43%, similarly, when LSTM is evaluated with the DIARETDB 1 dataset, it obtains a classification accuracy of 97.39%. Thus, the LSTM classifier with LBACS segmentation achieves better results due to an effective segmentation performed by LSIMBIF and AACM. Figures 7, 8 show the graphical representation of the performance of the classifier for different datasets such as IDRiD and DIARETDB 1. The LSTM classifier used in this research have the ability to capture long term dependencies and the complex patterns of the image. The LSTM generates visual captions for the image without vanishing gradient problem which helps in an effective classification and provides better classification results.

Moreover, the LBACS-LSTM is evaluated with various k-fold values such as 1,3,5,8 and 10. Table 6 and Table 7 present the analysis of the proposed approach for different K-fold values.

**TABLE 6:** Evaluation of LBACS-LSTM for IDRiD dataset with different K-fold values.

K-values	Accuracy (%)	Specificity (%)	Sensitivity (%)	Precision (%)
1	93.49	91.47	90.52	93.30
3	94.72	93.35	94.86	92.55
5	99.43	98.85	98.37	99.55
8	96.14	94.49	95.18	98.32
10	95.17	96.52	94.73	97.73

**TABLE 7:** Evaluation of LBACS-LSTM for DIARETDB 1 dataset with different K-fold values.

K-values	Accuracy (%)	Specificity (%)	Sensitivity (%)	Precision (%)
1	92.09	89.81	90.77	97.14
3	95.56	96.44	94.75	95.35
5	97.39	96.55	98.91	97.12
8	95.07	94.34	96.44	92.05
10	96.67	97.57	97.29	94.07

The results from Table 6 and Table 7 depict that the proposed approach achieves better value of accuracy, specificity, sensitivity, and precision for IDRiD and DIARETDB 1 datasets. The LBACS-LSTM achieves better metrics when the K-value is assigned as 5, when the K fold is assigned as 5, the data is separated in the ratio of 80% for training and 20% for testing.

After the evaluation of proposed approach with different K-fold methods, the memory usage of the proposed approach is evaluated for training. The Table 8 depicted below shows the memory usage of the proposed approach for different two different datasets such as IDRiD and DIARETDB 1.

**TABLE 8:** Evaluation of memory usage for different K-values for LBACS-LSTM.

K-values	Dataset	Memory usage (KB)
1	IDRiD dataset	122
3		168
5		173
8		194
10		208
1	DIARETDB 1 dataset	147
3		182
5		218
8		236
10		254

The results from the Table 7 shows that the suggested approach utilized 122 kB of memory when the k-value is assigned as 1 for IDRiD dataset and 147 KB, of memory for the same k-value of 1. When the value of k is 10, the proposed approach utilized memory of 208 KB, for IDRiD dataset and 254 KB, for DIARETDB, 1 dataset when the k value is assigned as 10.

Next to the evaluation of memory usage, the scalability of the dataset is evaluated when the proposed method is evaluated with 20%,40%,60%,80% and 100% of the data obtained from IDRiD dataset and DIARETDB 1 dataset. The existing classification approaches such as RNN, CNN, GRU and GAN are used to evaluate the performance of the LSTM classifier used in this research. The performance is evaluated based on the classification accuracy as the evaluation metric. The Table 9 depicted below presents the results obtained while evaluating the proposed approach.

**TABLE 9:** Evaluation of classification accuracy for varying data usage.

Data usage (%)	Models	Classification accuracy (%)	
		IDRiD	DIARETDB 1
20	RNN	91.09	89.69
	CNN	89.22	87.54
	GRU	88.74	92.10
	GAN	90.34	86.76
	LSTM	94.48	93.25
40	RNN	92.72	90.25
	CNN	90.29	87.25
	GRU	89.88	93.46
	GAN	91.45	88.19
	LSTM	96.22	94.76
60	RNN	93.17	91.67
	CNN	91.82	88.12
	GRU	95.69	94.21
	GAN	92.54	89.41
	LSTM	97.67	95.22
80	RNN	94.45	92.33
	CNN	92.18	89.87
	GRU	96.55	95.42
	GAN	93.12	90.14
	LSTM	98.75	96.64
100	RNN	95.23	93.23
	CNN	93.64	90.75
	GRU	97.45	96.52
	GAN	94.89	91.87
	LSTM	99.43	97.39

The results from the Table 8 shows that the LSTM classifier utilized in this research obtained better classification accuracy when compared with other existing classification approaches. For instance, when 20% of the data is used, the classification accuracy of the proposed approach is 94.48% whereas the existing classification approaches such as RNN, CNN, GRU and GAN obtained the classification accuracy of 91.09%, 89.22%, 88.74% and 90.34% respectively. The better result of the LSTM classifier is due to the effective segmentation performed using the proposed LBACS which effectively segments the DR images in a precise manner by considering the boundary levels.

**FIGURE 9:**
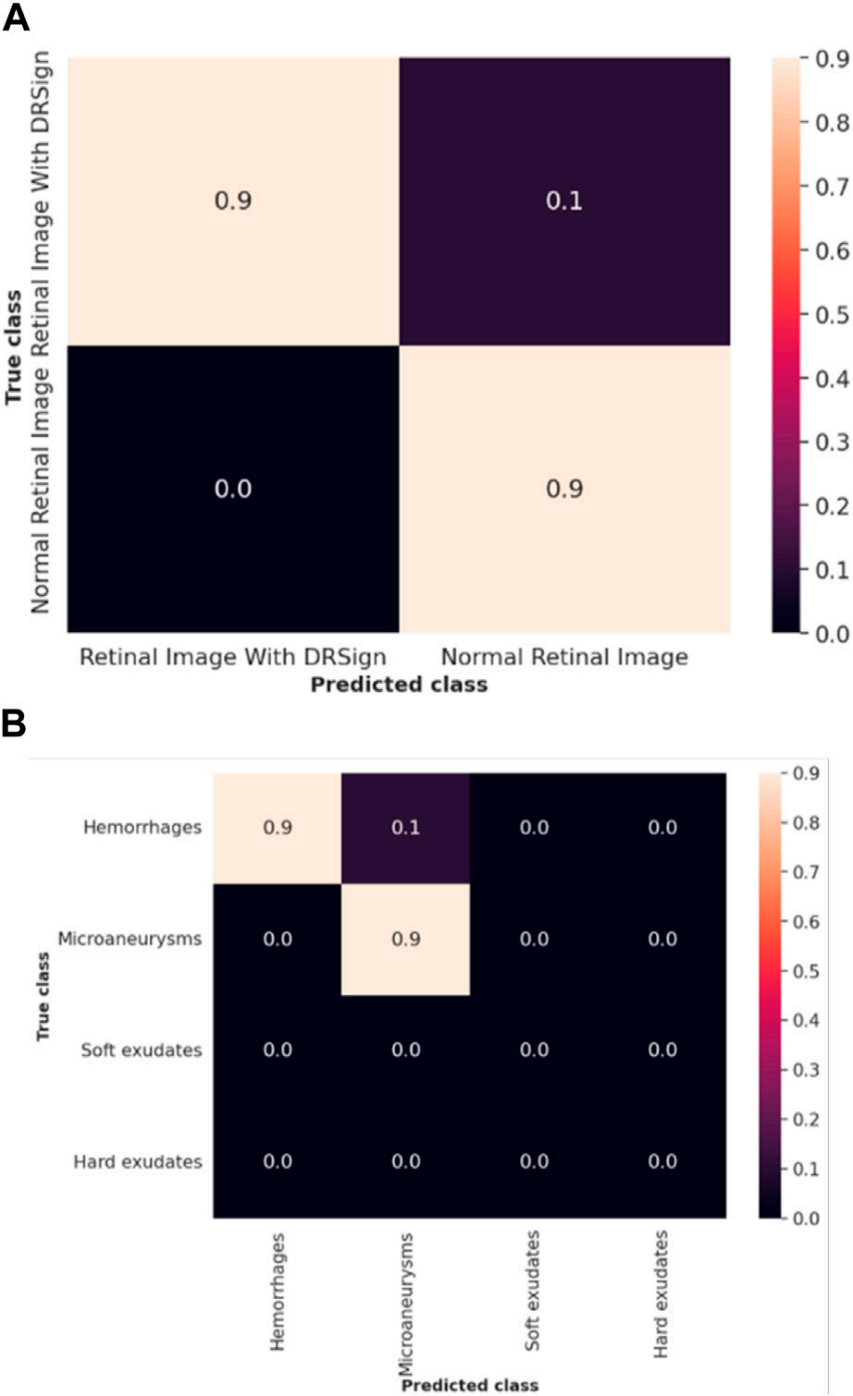
Confusion matrix of **(A)** IDRiD dataset **(B)** DIARETDB 1.

**FIGURE 10:**
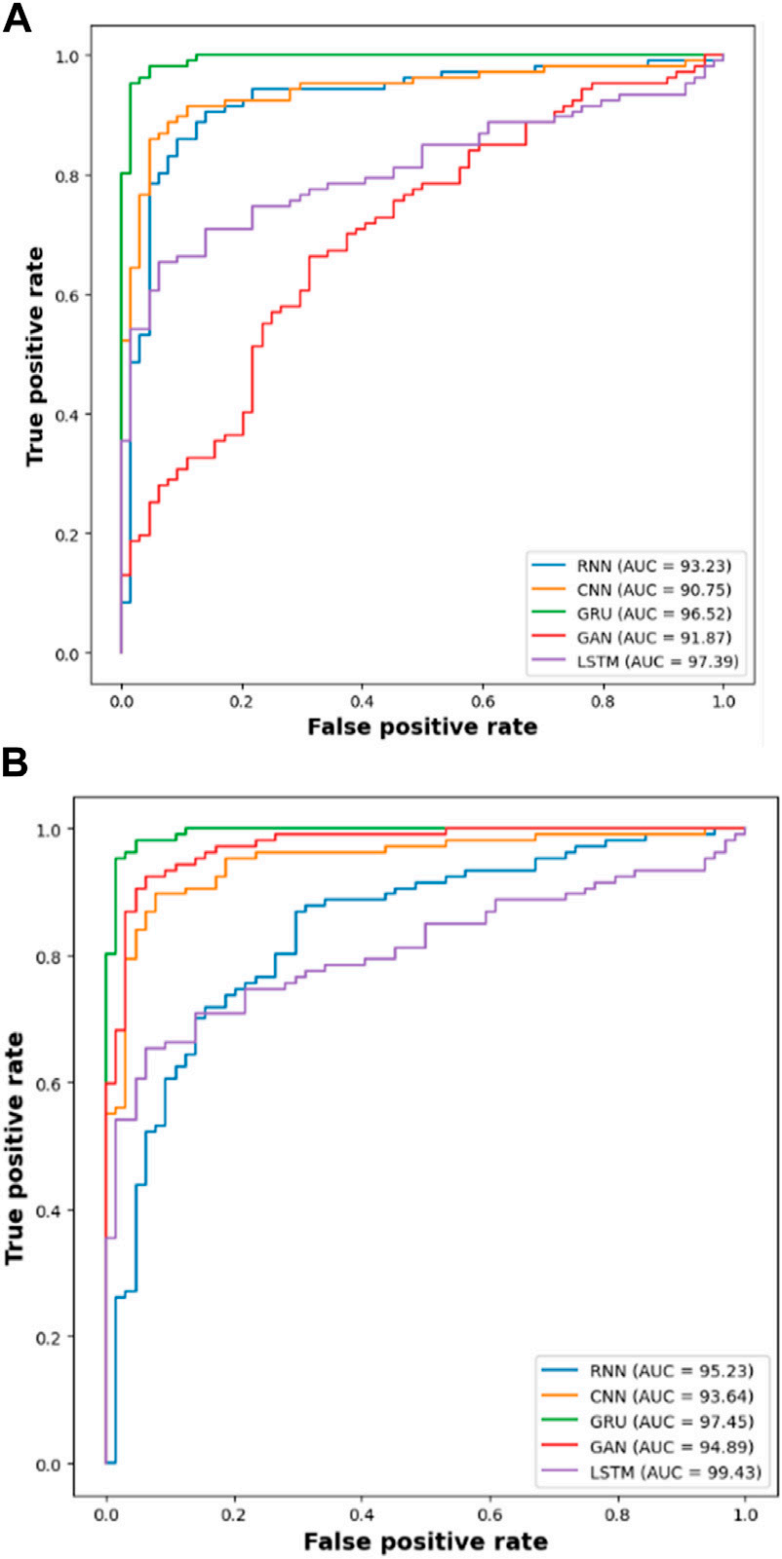
ROC of **(A)** DIARETDB 1 and **(B)** IDRiD 1.

The efficacy of the LSTM classifier used in this research is determined with the help of the confusion matrix and Receiver Operational Characteristics (ROC) curve which are shown in Figure 9 and Figure 10 respectively. The ROC graph depicts the measure of classification performance and it is graphically represented in True Positive Rate (TPR) and True Negative Rate (TNR).

### 4.4 Comparative analysis

The comparison of proposed LBACS-LSTM with the existing approaches such as three-dimensional semantic model (Jebaseeli et al., 2019), KNN (Rachapudi et al., 2023), Computer-aided method (Shaukat et al., 2022), CTSA-SAE (Dayana and Emmanuel, 2022a; Dayana and Emmanuel, 2022b), DS-KL (Mondal et al., 2023), ESOA optimized hybrid RCNN-BiGRU (Alajlan and Razaque, 2023) and Adaptive CNN (Math and Fatima, 2021) are described in this section. Table 10 presents the comparative analysis for IDRiD dataset and Table 11 depicts the comparative analysis of DIARETDB 1 dataset.

**TABLE 10:** Comparative analysis for IDRiD dataset.

Performances (%)	Three-dimensional semantic models Jebaseeli et al. (2019)	DS-KL Mondal et al. (2023)	ESOA optimized hybrid RCNN-BiGRU Alajlan and Razaque (2023)	LBACS-LSTM
Accuracy	97	97.67	99	99.43
Sensitivity	DNA	92.45	98.4	98.85
Specificity	98	DNA	98.6	98.87
Precision	87	DNA	98.5	99.55

**TABLE 11:** Comparative analysis for DIARETDB 1 dataset.

Performances (%)	KNN Rachapudi et al. (2023)	Computer aided method Shaukat et al. (2022)	CTSA-SAE Dayana and Emmanuel. (2022b)	Adaptive CNN Math and Fatima. (2021)	LBACS-LSTM
Accuracy	95	96.78	95.48	DNA	97.39
Sensitivity	92.6	92.47	93.29	96.37	96.55
Specificity	87.56	DNA	91.89	96.37	96.49
Precision	96.09	DNA	DNA	DNA	97.12

The results from Table 10 and Table 11 show that the proposed LBACS-LSTM achieves better performance in overall metrics when compared with the existing three-dimensional semantic models (Jebaseeli et al., 2019), KNN (Rachapudi et al., 2023), Computer-aided method (Shaukat et al., 2022), CTSA-SAE (Dayana and Emmanuel, 2022b), DS-KL (Mondal et al., 2023) and ESOA optimized hybrid RCNN-BiGRU (Alajlan and Razaque, 2023) and Adaptive CNN (Math and Fatima, 2021). The accuracy of the proposed method for the IDRiD dataset is 99.43% and 97.39% for the DIARETDB 1 dataset. The data which is not available is represented as DNA (i.e., Data Not Available). The better result of the proposed approach is due to the effectiveness of the suggested method by improving the boundary condition of the pre-processed image using LSIMBIF, and the AACM detects the edge of the image obtained from LSMIBIF, and also minimizes the size of visual data and aids in better classification.

### 4.5 Discussion

This research is carried out by considering a precise segmentation and classification of diabetic retinopathy. The proposed LBACS-LSTM is evaluated with two datasets, namely, IDRiD and DIARETDB 1. The performance of the proposed segmentation model is compared with evaluated based on segmentation accuracy and dice co-efficient. The existing segmentation models such as FCM, RG, LSM and ACM are used in comparing the performance of the proposed segmentation model. For instance, by considering IDRiD dataset, the segmentation accuracy of proposed approach is 98.87% whereas the existing FCM, RG, LSM and ACM obtains segmentation accuracy of 98.52%, 96.79%, 96.09% and 97.24% respectively. The performance of the LSTM classifier used in this research is evaluated with the existing classification approaches such as RNN, CNN, GRU, and GAN. The DIARETDB 1 dataset is used to evaluate the performance of the classifier. The LSTM classifier obtains the classification accuracy of 97.39% whereas the existing RNN, CNN, GRU and GAN obtains classification accuracy of 93.23%, 90.75%, 96.52% and 91.87% respectively. In a comparative analysis, the proposed approach is evaluated with three-dimensional semantic model and DS-KL for the IDRiD dataset. In the same way, the proposed method is evaluated with KNN, computer-aided method and CTSA-SAE for DIARETDB 1 dataset. The LBACS-LSTM obtains an overall accuracy of 99.43% for IDRiD dataset, whereas the existing three dimensional semantic model and DS-KL obtain overall accuracy values of 97% and 97.67%. When the proposed method is evaluated with DIARETDB 1 dataset, it obtains an overall accuracy value of 97.39%. The value of LBACS-LSTM for the DIARETDB 1 dataset is comparably higher than KNN, computer aided method, and CTSA-SAE with accuracies of 95%, 96.78%, and 95.48% respectively. The better result is due to the effectiveness of the suggested methodology by improving the boundary condition of the pre-processed image using LSIMBIF and the AACM. The existing approaches were incapable to detect the edges of the pre-processed image and aids in poor segmentation accuracy. But the proposed approach effectively segments the image into partitions and detect the DR in every individual segments. This process effectively enhances the performance of segmenting the images including edges of the images. Moreover, the suggested approach detects the edge of the image obtained from LSMIBIF, and minimizes the size of visual data which further minimizes the complexity while classifying the DR images.

## 5 Conclusion

In this research, diabetic retinopathy segmentation and classification are performed to segment and categorize various classes of diabetic retinopathy. The LBACS is proposed to perform an effective segmentation which helps to diminish the complexities while segmenting the images. After data acquisition, pre-processing is performed for the removal of noise, enhancing the color and luminosity of the image. Then, the proposed LBACS is used to segment the pre-processed image using LSIMBIF with the improved boundary condition and the AACM is utilized in the process of detecting the edges of the image obtained from LSMIBIF, while minimizing the size of visual data and aiding in better classification. The features are extracted using GLCM, HOG and LTP, finally the various classes of diabetic retinopathy are categorized with the help of LSTM. The accuracy of the proposed LBACS-LSTM for IDRiD and DIARETDB 1 datasets is 99.43% and 97.39% respectively. Similarly, the sensitivity of the proposed approach is 98.85% for the IDRiD dataset and 96.55% for the DIARETDB 1 dataset. However, the segmentation accuracy of the proposed approach is minimized when the images with extreme noises are subjected as input.

### 5.1 Future scope

The experimental results show that the proposed approach obtains better results by means of segmentation and classification. However, the absence of feature selection probably diminishes the overall performance of the model in classifying DR images. So, the optimization based feature selection will be performed in future to obtain better classification results.

## Figures and Tables

**Table:** 

**Parameter**	Description
$g\left(x,y\right)$	Gaussian function
$I_{g}\left(x,y\right)$	Green channel component
$I_{g}\left(x,y\right)$	Gaussian noise
$g^{\prime }$	Edge indicator function
μ,λ,α	weighted coefficient values
$d_{p}$	double well potential distance
$\nabla_{\phi }$	differentiation of the of initialization process
$\delta_{\varepsilon }$	hat function along variable support
ϕ	zero level set of level set factor
$\Omega_{in}$	level set factor
$\Omega_{0}$	zero-level set
$E\left(\phi \right)$	energy function
$\varepsilon_{img}$	the external energy which is determined with the help of image attribute
σ	standard deviation
$I_{\sigma }$	Smoothened image
ρ	Boundary threshold function
$\frac{\partial \phi }{\partial t}$	The difference in level set factor with respect to time
∇	gradient operator
$\delta_{\varepsilon }$	Dirac function
$E_{R}$	data fitting term
$E_{D}$	Data regularization term
$P_{y,\Omega_{x}}$	intensity of distributing small circular neighbourhoods
θ	Normalization factor
${\hat{I}}_{\phi_{i}}\left(y\right)$	fitting intensity value
$I_{\phi_{i}}\left(y\right)$	real intensity value
$u_{i}^{m}\left(x\right)$	dependent membership function
$b\left(x\right)$	varying bias field related to inhomogeneous intensity
$n\left(y\right)$	Gaussian distribution function
ϕ	smoothened level set function
$p\left(\left|\nabla \phi \right|\right)$	energy density function
Pc	central pixel value
τ	threshold value
Pi	Neighboring pixel value
$G_{X}$ **and** $G_{Y}$	gradient values of X and Y
$h_{t-1}$	hidden state of the prior layer
$x_{t}$	input for the current layer
Wandb	weight and biased state
$f_{t}$	Forget gate
${\tilde{C}}_{t}$	Intermediate Temporary state
$o_{t}$ **and** $h_{t}$	output gate and the hidden state of the succeeding layer
**Description**	Abbreviation	
**2D-SWT**	2-dimensional stationary wavelet transform	
**ACM**	Active Contour model	
**AACM**	Adaptive-Active Counter Model	
**CTSA**	Chronological Tunicate Swarm Algorithm	
**CFP**	Colour Fundus Photographs	
**CTSA-SAE**	Computer aided method and Chronological Tunicate Swarm Algorithm with Stacked Auto Encoder	
**CLAHE**	Contrast Limited Adaptive Histogram Equalization	
**CNN**	Convolutional Neural Network	
**DNN-BOA**	Deep Neural Network and Butterfly Optimization Algorithm	
**DSA-KL**	Delimiting Segmentation Approach Using Knowledge Learning	
**DS-KL**	Delimiting Segmentation Approach Using Knowledge Learning	
**DIARETDB 1**	Diabetic Retinopathy Database 1	
**DR**	Diabetic Retinopathy	
**DRLSE**	Distance Regularized Level Set Evolution	
**ESOA-HGRU**	Egret Swarm Optimization based Hybrid Gated Recurrent Unit	
**FCM**	Fuzzy C Means	
**GAN**	Generative Adversarial Network	
**GLCM**	Gray Level Co-occurrence Matrix	
**IDRiD**	Indian Diabetic Retinopathy Image Dataset	
**KNN**	K-Nearest Neighbor	
**LSMIBIF**	Level Set Method with Improved Boundary Indicator Function	
**LBACS**	Level-set Based Adaptive-active Contour Segmentation	
**LSTM**	Long Short-Term Memory	
**MPA**	Marine Predictor Algorithm	
**MEM**	Modified Expectation Maximization	
**ML-FEC**	Multi-Label Feature Extraction and Classification	
**PBIL**	Population-Based Incremental Learning	
**ROC**	Receiver Operational Characteristics	
**RNN-LSTM**	Recurrent Neural Network-Long Short-Term Memory	
**RDRN**	Refined Deep Residual Network	
**RG**	Region growing	
**S-CNN**	Segmentation Convolutional Neural Network	
**TNR**	True Negative Rate	
**TPR**	True Positive Rate	
**TSSMO**	Tunicate Swarm Spider Monkey Optimization	

## Data Availability

The original contributions presented in the study are included in the article/supplementary material, further inquiries can be directed to the corresponding author.
